# Structural insights from water-ferredoxin interaction in mesophilic algae and halophilic archaea

**DOI:** 10.6026/97320630015079

**Published:** 2019-02-28

**Authors:** Amal Kumar Bandyopadhyay, Rifat Nawaz U Islam, Debanjan Mitra, Sahini Banerjee, Arunava Goswami

**Affiliations:** 1 Department of Biotechnology,The University of Burdwan,Burdwan, West Bengal,India; 2 Department of Zoology,The University of Burdwan,Burdwan,West Bengal,India; 3 Department of Biological Sciences,ISI,Kolkata,West Bengal,India

**Keywords:** Bound-waters, interior bound waters, cavity bound waters, bridge interactions, halophilic signature, ferredoxin recognition

## Abstract

We analyzed the water-ferredoxin interaction in mesophilic (moderate temperature) algae (PDB ID: 1AWD) and halophilic (salt-tolerant) archaea (PDB ID: 1DOI) using POWAIND version 2.0 (a protein-water interactions calculation program). It is found that the shell water (SW) is 2.5 fold greater in halophilic ferredoxin than mesophilic ferredoxin. Water-ferredoxin interactions in the core and cavity are the signature of stability. The normalized frequency of such interactions is less in halophilic relative to mesophilic ferredoxin and the halophilic signature for stability by such interactions is negligible. However, the surface dominated with such interactions seems to be important for ferredoxin and oxido-reductase recognition.

## Background

Primary structures of mesophilic protein form the native state in aqueous environment [1]. A quite surprising observation was entertained in the case of halophile [2] in that the ferredoxin from Halobacterium salinarum retains its native state only in saturated brine (≥4.5M NaCl) conditions [3]. In general, the protein structure maintains layers of hydration shells [4]. Upon crystallization, although bulk waters are largely eliminated, shell-waters (SWs) remain bound to protein [5]. As these SWs are interacting with different a] atom-types and b] segments of secondary structure and c] regions such as core, surface, and cavity of 3D-structure of protein [5,6], detailed understanding of these interactions may illuminate the role of SWs for the maintenance of the structure, stability, recognition, and function of proteins. The role of SWs largely depends on its location in the protein molecule. While SWs in the core and cavities are directly related to the stability and function, surface-bound-waters are related to the binding specificity of protein. Notably, in the core of the protein, destabilizing isolated charged-atoms, hydrogen-bond donor or acceptor groups and flexible arrangements of secondary structure are largely satisfied by SWs and thereby contribute to the overall stability of protein [5,6]. Internal cavities could be filled by ordered or disordered SWs. While in crystal structures, internal polar or hydrophobic cavities are seen to be occupied by ordered SWs [5,6,7,8], disordered SWs are detected in the hydrophobic cavities in NMR elucidated structures [6]. As far as interactions between protein and SWs are concerned, hydrogen bond, electrostatic, van der Waals interactions play a major role [9]. Broadly, the distance between atom types of protein (ATPs) and SWs vary between 2.5Å - 4.5Å. These interactions could either be isolated-type i.e. SW: ATP is 1:1 or bridged-type i.e. SW:ATP or ATP:SW is 1:n (n≥2) [5,6]. While polar atom-type of protein (PATP) makes strong or weak hydrogen bonds or electrostatic (salt-bridges or ion-pairs) interactions, non-polar atom-type of protein (NPATP) makes van der Waals (vdW) interactions.

Detection of ordered water in the core or internal cavity [10] needs the knowledge of B-factor and occupancy [11,12]. Lower the Bfactor, lesser is the fluctuation of atoms and thus, it is easier to locate them at a given position. In other words, at high resolution (~0.2nm), the B-factor is low and thus, interacting atoms are readily located. Ordered SWs that bind ATPs in the cavity or core of the protein has a lower degree of freedom and motion. Such PATPs/NPATPs possess low B-factor [5,11]. The core of protein is dominated by a segment of secondary structure (helix/strand) [13], simply because the amino acids that have a high propensity for the β-strand (VAL, ILE, PHE) and α-helix (ALA, LEU, MET) are hydrophobic. It has been shown that the internal SWs form bridge interactions with secondary structures (H/S/C) and thereby contributing to the overall stability of protein [8]. Although great deals of work on SW-ATP interactions have been achieved, more remains to be addressed. The question as to does there exist binding preference of SWs for a given segment (H/S/C) and location (core/surface i.e. b/e) of protein remained to be worked out. How these preferences modulate the stability of proteins operating under normal vs extreme environment? Orthologous proteins functioning under diverse environmental conditions (such as ferredoxin from mesophilic algae i.e. 1AWD from Chlorella fusca and halophilic archaea i.e. 1DOI from Haloarcula marismortui) may have differential features for the above-mentioned properties. It is worth mentioning that the water activity in which mesophilic proteins function almost equals to one, which is highly detrimental for the functioning of halophilic proteins [2,3,14]. The latter, in turn, functions at a saturated salt solution (≥4.5M NaCl) in the cytoplasm, where the water activity is almost half of the mesophiles.

We present a fully automated procedure with many new features than the earlier version, which could be useful for the extraction of the above-mentioned attributes of SW-ATP interactions. Comparative analyses on SW-ATP interactions of 1DOI and 1AWD and understanding of the halophilic signature in the former have been the major focus of the present study. Temperature factor and occupancy related orderness and disorderness of SWs interacting with ATPs have also been highlighted in this work. Taken together, we address the effects of the environment on SW-ATP interactions, which we believe would be useful in protein engineering and structural bioinformatics.

## Methodology

### Datasets

High resolution, monomeric crystal structure of [2Fe-2S]-ferredoxin from mesophilic cyanobacteria (Chlorella fusca), and halophilic archaea (i.e. Haloarcula marismortui) are selected from Research Collaboratory for Structural Bioinformatics (RCSB) protein data bank (PDB) [16]. These two proteins are such selected that their topologies are similar but primary sequences are different (identity ≥30%). Some of the sequence and structure properties that are procured from the PDB database could be found in Table 1.

### Identification of buried/exposed and cavities ATPs of protein

In 3D structure, ATPs and the SWs could be found in different locations (b/e). Buried ATPs are identified by setting the limit of relative accessible surface area (RASA) of atoms as 22 Å^2^. ATPs having a RASA≤22 Å^2^ are taken as buried, otherwise exposed. The folded or absolute accessibility of ATPs are determined using the analytical procedure of accessibility [10]. The RASA of atoms is computed by using the accessibility of the folded [10] and unfolded states [18]. The following formula is used to compute the RASA of the atoms of the protein.

 RASA of ATP =ATP's accessibility in folded state *100/ATP's accessibility in unfolded form 

Further, cavities of the proteins are readily identified from the atomic accessibility output file "XXXX.txt" by the program, surface racer. These are interior cavities that are inaccessible to the outer solvent [10].

### Atomic details on a segment of the secondary structure

ATPs interacting with SWs may belong to different segments of secondary structure (H/S/C). Is there a preference of water atoms for a given segment? To work out this, we have extracted experimental results of helices and strands directly from the RCSB PDB files, by the use of POWAINDv2.0. The program assigns segments as H (helix), S (strand) and C (coil) on ATPs.

### Extraction of temperature factor and occupancy

Column 55-60 and column 61-66 of RCSB PDB file are for atomic occupancies and temperature factors respectively. These atomic details of SWs are directly extracted by POWAINDv2.0 to understand their orderness/disorderness and mobility.

### Determination of details on SW-ATP interactions

All inter-atomic and bridge interactions are computed as earlier in the range of distance specific manner [19]. Interaction is considered favorable when it is greater than 75% of the sum of van der Waals radii of interacting atoms. The interaction is otherwise marked as unfavorable. In ATP-SW interactions, the atoms of protein are classified as earlier e.g. side-chain polar atoms, main-chain O-type and N-type atoms, C-type and other non-polar atom-types as earlier [19].

### Automated extraction of information

Extraction of i] RASA of ATPs, ii] the location of helix (H), strand (S) and coil (C) of ATPs, iii] distance-range specific ATP-SW interactions, iv] interactions by internal-cavity bound waters, v] different types of bridge interactions in association with b/e and H/S/C information are made fully automated in POWAINDv2.0. The program needs X-ray crystal structure as input and the installation of surface-racer [9] in the defined directory for its functioning. The program is a higher version of POWAINDv1.0 [19], which keeps most of the earlier tasks along with additional features.

## Results

### General characteristics of the proteins

Plant-type [2Fe-2S]-ferredoxin takes part in wide variety of electron transfer reactions [20]. While 1AWD participate in photosynthesis in partially membrane-bound form [21], 1DOI acts as co-factor for oxidative decarboxylation reaction in the cytoplasm of halobacteria [2,4]. Cellular environments and solvent conditions in which ferredoxin functions differ drastically in that while 1AWD operates under mesophilic conditions, 1DOI works in high salt (≥4.5M) solutions. How the physicochemical and sequence properties of these proteins vary? To check these, we have analyzed physicochemical and sequence properties [22,23], substitution parameters [24], salt-bridge [25,26] and its energetics [27,28], and core and surface compositions (Table 3) [29], few of which are presented in Table 2. Several points are noteworthy. First, halophilic ferredoxin (hFD) is longer than cyanobacterial ferredoxin (cFD). Second, the hydrophobic and hydrophilic compositions of sequences of hFD and cFD are almost similar. The net negative charge follows the order as hFD>cFD. Third, the aliphatic Index, which is the indicator of the stability of protein [30], is seen to be greater than the threshold for both the ferredoxins. Forth, ferredoxin is highly acidic in nature in general with hFD is seen to be more acidic than the cFD. Fifth, the difference in homologous positions of hFD and cFD is 61.7% [23] (Table 1). Sixth, both these ferredoxins have hydrophobic (HB) and hydrophilic (HL) residues in the core and surface of their tertiary structures [29]. Substitution analysis shows that NCS:CS follows the order as 1AWD»1DOI. Seventh, unlike 1AWD, in 1DOI out of 4 salt-bridges, two are in the core with one is inter-helix (HH) and other is inter-stand (SS) types (Table 2). The net-stability (ΔΔGnet) is higher in 1DOI (-14.7 kcal/mol) than 1AWD. Overall, results show characteristic differences in sequence, structure, and stability of these ferredoxins.

### Halophilic ferredoxin has a similar level of polar interaction as its mesophilic homologue

**Figure 1:**
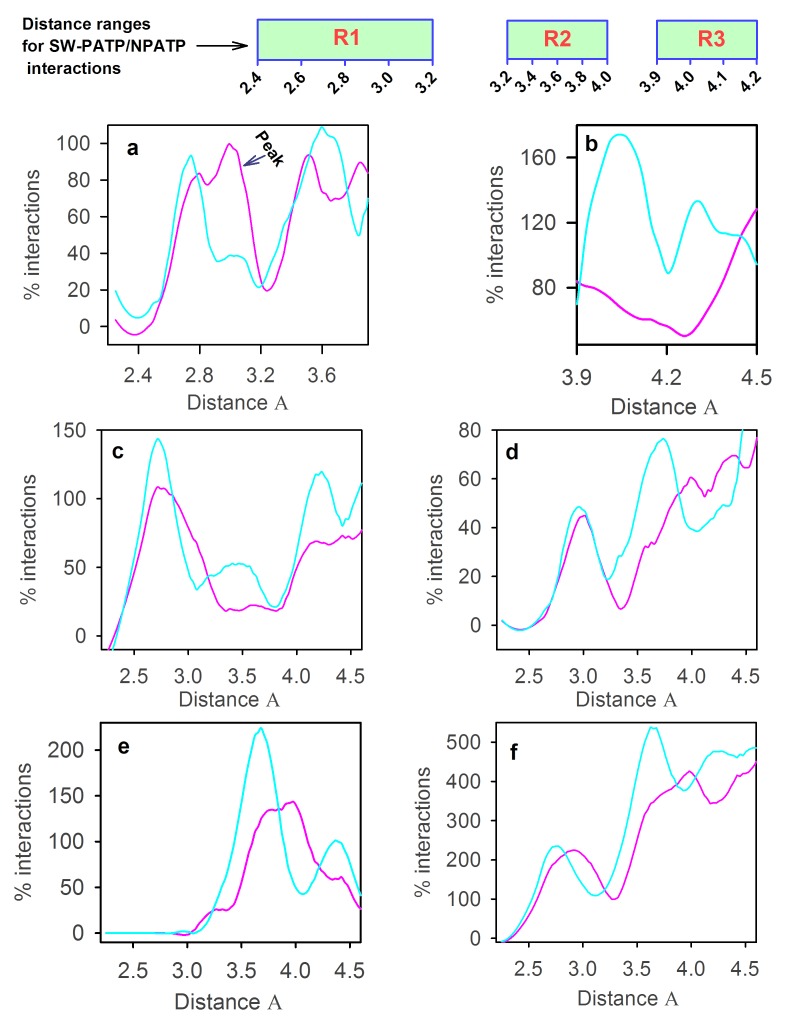
Plot of normalized frequency (in %) vs distance between the SW and (a) polar short-ranged, (b) polar long-ranged, (c) main-chain O-type atom, (d) main-chain N-type atom, (e) main-chain C-type atom and (f) all other non-polar atoms for 1AWD (cyan), and 1DOI (pink). SW = Shell-water; PATP = Polar atom type of protein; NPATP = non-polar atom type of protein.

It is of interest to know the difference in SW-ATP interactions in these ferredoxins. This is relevant as these functionally and topologically identical ferredoxins, which belong to different domains of life, are functioning under different solvent conditions. Protein is made up of twenty standard amino acids. Each amino acid has PATPs and NPATPs. The primary sequence of the protein is formed by main-chain and side-chain. O-type and N-type (20 for 20 amino acids) PATPs are found in the main-chain. There are 20 PATPs for the side-chains of amino acid. These are OD1, OD2 (ASP), OE1, OE2 (GLU), OD1, ND2 (ASN), OE1, NE2 (GLN), OG (SER), OG1 (THR), OH (TYR), NZ (LYS), NH1, NH2, NE (ARG), ND1, NE2 (HIS), NE1 (TRP), SD (MET) and SG (CYS). Main-chain contains two NPATPs, C and CA. Apart from these, side-chain of all amino acids contains a total of 67 NPATPs. All these ATPs of protein may interact with SW for its function and dynamic in the cell [31]. As these ferredoxins possess an uneven number of SWs (Table 1), the normalized plot is shown for different distance ranges (Figure 1; R1, R2, and R3). Several points are noteworthy from the figure. Since the length and net negative charge of the sequence of 1DOI is higher (Table 2) and since the net negative charge on the surface of the protein is also higher than 1AWD (Table 3), we anticipated much greater level of interactions of SWs with PATPs (Figure 1a and 1b) in 1DOI than 1AWD. Although 1DOI has a higher level of SW- PATP interactions (for peak region; Figure 1a), it is almost comparable with that of 1AWD, for other regions (Figure 1b-f). In the strong hydrogen bond region (2.4Å- 3.2Å; R1), although 1DOI has a strong peak for SW-PATP interactions around 3Å, it is lowered in the weak hydrogen bond region (3.2Å - 3.9Å i.e. R2; ~3.6Å). Further, the level of SW-PATP interactions of 1AWD at ~4Å is much higher than 1DOI (Figure 1b).

Although the level of SW-PATP interactions in 1DOI and 1AWD vary to some extent for different polar regions (2.4Å-3.2Å i.e. R1, 3.2Å - 3.9Å i.e. R2 and 3.9 Å-4.2Å i.e. R3), the resultant level of interactions of these proteins is seen to be similar.

It is seen that 1AWD dominates over 1DOI for both O-types (Figure 1c) and N-types (Figure 1d) of interactions. Interestingly, while in O-types (Figure 1c), the regions R1, and R3 are seen to be contributing, in N-types, three regions R1, R2 and R3 are making contributions in SW-PATP interactions. Notably, for the first region (R1), the level of O-types mediated SW-PATP interactions is more intense than that of the N-types. Taken together, 1AWD dominates over 1DOI in these region-specific interactions. Non-polar atom types (NPATPs) also interact with SWs. The main-chain C-type atoms are seen to make strong NPATP-SW interactions for the R2 region. Here also, the 1AWD dominates over 1DOI (Figure 1e). The plot of all other NPATP-SW interactions is shown in Figure 1f. These atom-types are made of α, β, δ, ε, η and ζ carbon atoms from the side-chains. Surprisingly, it is seen in Figure 1f that there is a satellite-peak of SW-NPATP interactions for the R1 region and also dominant ones at R2 and R3, in which 1AWD dominate over 1DOI. Taken together, it is seen that both PATPs and NPATPs interact with SWs largely for three regions. In all these cases, 1AWD is seen to dominate over halophilic ferredoxin (1DOI).

### Preference of SW on helix/strand/coil and core/surface atom-types

The 3D structure of ferredoxin falls in the (α+β)-SCOP (structural classification of protein) class. The fold of the protein is in the β- grasp (ubiquitin-like) super-family. In the 3D structure, residues are present in different segments of secondary structures. Further, sidechains of residues could either be buried or exposed. These secondary structures with differential accessibility form the folded protein a globular in shape. Here, although surface and core compositions (Table 3) show wide variations for these ferredoxins, their main-chain topologies remain almost similar (RMSD ~1.5Å). It is of interest to know the preference in SW-ATP interactions, of a given ATP in a given region (b/e) and segment (H/S/C). It is also of interest to know the orderness and disorderness of SWs for such a preference, if any.

**Figure 2:**
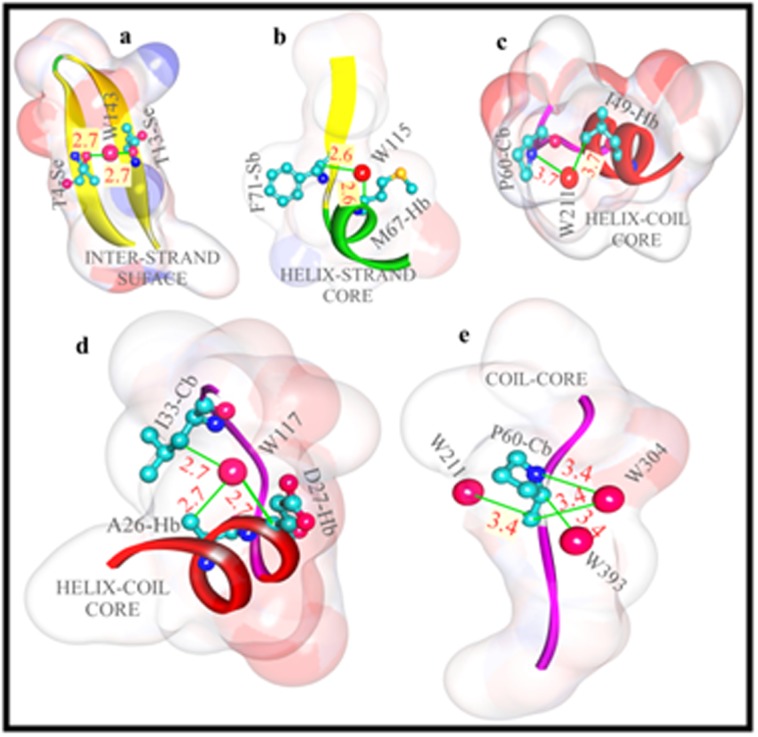
Typical plot of different types of inter-segment P:W type bridge interactions, such as inter-strand (a), helix-strand (b), strand-coil (c), helix-coil multiple types (d). A W:P type bridge is also shown (e), which is intra-coiled type.

To check this, we have classified SW-ATP interactions into 6 categories (Cb, Ce, Hb, He, Sb and Se) for R1, R2 and R3 regions. For example, Cb implies that the ATP is in Coil and in the core of the protein. Similarly, Ce, Hb, He, Sb and Se also have a similar meaning. For each of the category, POWAINDv2.0 determines the frequencies of ATPs, temperature factors of SWs and accessibilities of ATPs, the result of which is shown in Table 4. Several points are noteworthy from the table. First, irrespective of domains of life (mesophilic or halophilic) and regions (R1, R2, and R3) of the interactions, the frequencies (Q) of Cb (ATP in coil and core) and Ce (ATP in coil and surface) dominate over Hb, Sb and He, Se respectively. Notably, amino acids with a propensity for helix and strand are more hydrophobic in nature than that in the coil segment. In other words, from the hydrophobicity point of view, these segments follow the order as strand>helix>coil [32]. The observation thus indicates that the SW-ATP interactions are mediated largely by PATPs with the coil segments. Second, buried ATP interacting with SWs for these regions (R1, R2, and R3), have much lower average temperature factors (TFav) than that of the surface. For example, in the case of 1AWD, the Cb has TFav as 23.0, 22.0 and 21.2 for R1, R2 and R3 regions respectively, which are 30.2, 27.1 and 29.2 respectively for the Ce class. It is the case for 1DOI (Table 4). Remarkably, it is noteworthy that the TFav for the 1DOI cases (both for the surface and the core) are seen to be at least 5 times lower than that of the 1AWD (Table 4). Third, the average RASA of ATPs, which are computed from accessibilities of atomtypes interacting with bound-waters in the core of the protein, are seen to be much lower (<12 Å^2^) than the standard threshold value (20Å^2^). Finally, all these classes (Cb, Ce, Hb, He, Sb and Se; (Table 4) involve inter/intra-helix, inter/intra-strand, coil-helix, coil-strand and coil-coil bridge interactions with SWs in the core and in the surface (Figure 2).

### Different forms of bridge interactions

Numbers of experimentally observed water molecules are 320 and 126 in 1DOI and 1AWD respectively. The bridge interactions are of two types, i.e. P:W=1:n and W:P=1:n, where P and W indicate ATP and SW respectively; n≥2. Here, we describe each ATP by two items, secondary structure (C/H/S) and accessibility (b/e). Hb thus indicates an ATP, which is in a helix and in buried conditions.

HbCe indicates two ATPs (bridge-partners) with one in a helix and in buried form and the other is in a coil and in exposed form. It has been reported that bridge interactions are directly related to the stability of protein [5,6]. It is more so when the interacting partners are present under buried conditions [5]. Buried waters are less mobile (low TF) and thus could contribute more to the overall stability of protein [5]. In the interior of proteins, unsatisfied charges, donor-donor or acceptor-acceptor proximity and flexibility of secondary structure segments are common, which could largely be circumvented by SW-ATP mediated bridge interactions [5,9]. To check this, we performed detailed investigations by the use of POWAINDv2.0, the results of which are presented in Table 5 and Figure 2. Following observations are noteworthy from the table and figure. First, although experimentally observed SWs are much higher in 1DOI, it has a much less normalized frequency of bridgeinteractions (both P:W and W:P types) than that of its mesophilic homologue (1AWD). It is seen that for R2 region (Table 5), while 1AWD has 79.8% and 56.4% of P:W and W:P ype of bridge interactions, 1DOI has only 53.1% and 47.7% respectively. Notably, halophilic proteins in general function under saturated salt solution [2], where the water activity is much lower than the mesophilic conditions [33]. Thus, it appears that although detected waters are at a much higher level in 1DOI compare to mesophilic ferredoxin, their participation in bridge-interactions seems to be completed by salt ions and thus making these interactions at low limit. Second, since buried waters are more important in terms of stability of proteins in general [5,6] and since stability is an issue for proteins functioning under extreme environments of high salt (1DOI), it is imperative to check buried bridge interactions for these proteins. Here, in both 1DOI and 1AWD, while P:W-type bridges (Figure 2e) are more of intra-segmental (CbCb/HbHb/SbSb) and local type, W:P-types (Figure 2a-d) in turn, are inter-segmental (inter-strand; Figure 2a) and long-ranged types such as CbHb (coiled-helix; Figure 2c), HbSb (helix-strand; Figure 2b) and CbSb (coiled-strand; Figure 2c), the normalized frequencies for these types of bridge vary greatly. It is seen that buried bridge interactions of P:W and W:P-types in 1DOI are only 9.3% and 12.5% for region R2 (Table 5), which in mesophilic ferredoxin (1AWD) are 22.3% and 21.3% respectively. It is worth raising the question here as to what is the force that may have replenished the deficit of low level of bridge interactions in 1DOI. Salt-dependent stability of halophilic ferredoxin [2,3], seems to be the prime contributors that take the care of the above-mentioned bridge energy gap. Third, based on the location (b/e) and secondary structure types (H/S/C), three types of bridge-interactions are possible: completely exposed (Figure 2a), half-exposed and fully buried. As far as secondary structures are concerned, any one of these types is seen to be associated in various combinations (Table 5). In 1AWD, inter-segmental and long-ranged types of bridge are more frequent than that of 1DOI. Forth, bridge interaction between SWs and ATPs can be of extended types, where more than two ATPs are involved (e.g. CbCbCbHe). Figure 2d shows such a typical bridge interaction. Here, three atom-types of Hb, Hb, and Cb are making interactions with an SW. In our study, with mesophilic, halophilic ferredoxins, we have observed 1:2, 1:3, 1:4, 1:5 and 1:6 types of bridge interactions (Table 5).

### Comparative analysis of internal cavities and its interaction with SW (≤5.5 Å)

3D structure of protein is the result of both favorable and unfavorable interactions, which makes the former energetically compromised to achieve the characteristic global minimal state. Packing of atoms in some regions of protein is not perfect but possesses cavities or unoccupied space, which are evidenced by NMR, X-ray crystallography and molecular dynamics simulation studies [6]. These purely internal or exteriorly connected internal cavities have an important role in the stability, flexibility, and functionality of proteins [34,8]. Internal cavities could be filled up with waters or other types of molecule or they could be empty as well [35,8]. The water-filled cavity can bring stability up to -12 Kcal/mol [36]. Cavities, clefts, and pockets are the terms that are used to describe the shape, size, and characteristics of these local spaces of protein [37]. Different types of analytical software are developed to detect crucial cavities from protein structures [37,10]. The software surface racer detects cavities that are inaccessible from the surface of proteins [10]. Functionally identical proteins are expected to have similar types of cavities as it has been found that functionally different but similar sized proteins vary greatly by the content and characteristics of cavities [8]. Cavity-waters making a number of interactions including their ordering around non-polar parts, hydrogen-bonds and van der Waals contacts with the constituent of the cavity and thereby restoring the folded state of the protein [38].

**Figure 3:**
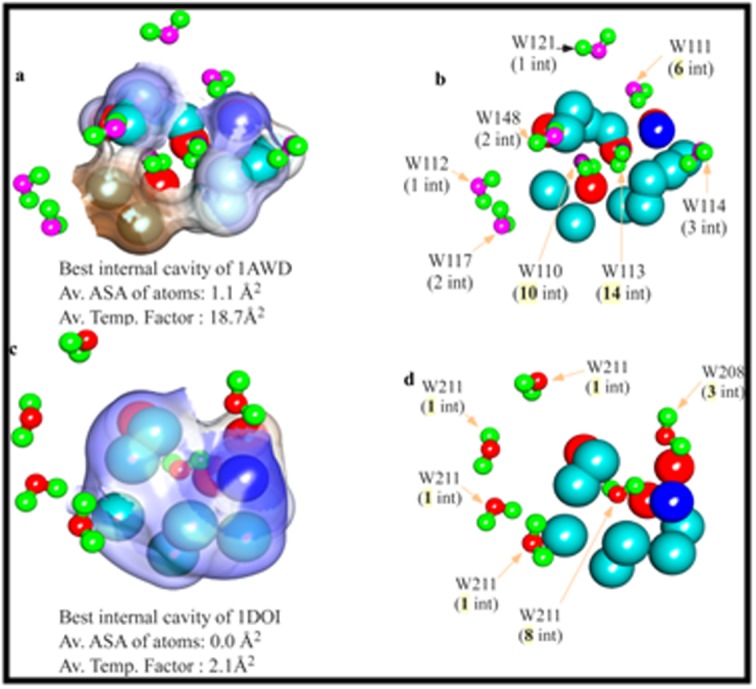
Two views of the best cavity of 1AWD (a and b) and 1DOI (c and d) that are filled with SWs. In left-side, the cavities of these proteins are shown with the soft-accessible surface (a for 1AWD, c for 1DOI), which is removed in the right-side for better visibility and identification of bound-waters (b for 1AWD, d for 1DOI).

POWAINDv2.0 based extraction of SW-ATP interactions shows a number of SWs are involved in these interactions around the cavities (Table 6). For example, in the case of 1AWD, a total of 8, 3, 4 SWs are interacting in cavity-1 (Cv-1), Cv-2 and Cv-3 respectively (Table 6). How many of these SWs are inside the cavity? Although the numbers of interior cavities are 3 in each of 1AWD, and 1DOI and although the number of interactions is also high, only one cavity from each of these proteins is seen to be internally filled with SWs (Table 6: pink shaded SWs and Figure 3a and c). Other SWs of cavities are seen to be present in the vicinity or in the crevices of the cavity (Figure 3a, c). For example, in cavity 1 (Cv-1) of 1AWD, there are 8 moles of interacting waters (Table 6) of which only two (i.e. W113 and w110) are present inside the cavity (Cv-1) that are making 14 and 10 SWs-ATPs interactions from inside (Figure 3a and b). W111, W114, and W148 are in the crevices and rests are on the surface (Figure 3a). In 1DOI, these interactions happen similarly but to some lower extent than 1AWD (Table 6). A general observation is that, SWs that are present inside the cavity-space making maximum interactions than the cleft-bound and surfacebound SWs. Remarkably the internally bound SWs show an average distance of SWs-ATPs interactions ≤4.2Å. The number of highest interactions of internally bound water is 14 (W113) and 8 (W211) for 1AWD and 1DOI respectively. The average temperature factors for these SWs are 18.7Å^2^ and 2.1Å^2^ respectively (grey shade; Table 6).

## Discussion

### Extra regions of 1DOI bind additional SWs

Functionally identical proteins of different species are adapted under different solvent conditions, which have a crucial role for the folding [1], stability [5,8], specificity [31] and functionality [5] of the former. While mesophilic ferredoxin (cFD; 1AWD) functions in aqueous solution [20,21], its halophilic homologue (hFD; 1DOI) requires at least 4.5M NaCl for the maintenance of structure and stability [2,3,14]. Withdrawal of salt from the medium produces a partially unfolded form of the protein [3,14]. Such salt-dependent unfolding involves a molten-globule like state [14], indicated an essential role of salt for the protein. The structure of 1AWD was solved at a very high resolution (Table 1). The resolution of 1DOI is 1.9Å. Detected SWs are much higher (320 moles) in 1DOI than that of 1AWD (126 moles). The reason for higher SWs in 1DOI may be related to its length [8,22]. It has some 24 residues extra at the Nterminal end, which is compositionally enriched with acidic residues that are known to bind more waters [39].

### 1DOI has some unique structural features

Although 1DOI is functionally identical as 1AWD, analysis using web-tools [23,29] shows it has unique sequence and structure features. While homologous positions of 1DOI show a sequence variation of 65%-70% from 1AWD, their core and surface compositions are almost identical (Table 3). It thus seems that the difference in the common part is counterbalanced by the insertion regions at N- and C-terminal of 1DOI [39]. The lower nonconservative (NCS) to conservative (CS) substitution ratio (0.42) in 1DOI is surprising, which may indicate that the substitution mechanism, in this case, is more decisive and restricted than 1AWD (NCS:CS = 0.66). As hydrophobic force is lowered under saturated salt environment due to low water activity [33], some alternate forms of weak force that are less affected by the presence of multimolar salt in the medium may have balanced the deficit in the case of 1DOI. In fact, it is seen that the design of salt bridges and their energetics, as extracted using web-tools [25,26,27,28], has been an additional contributor, at least by part, to the stability of 1DOI. In comparison to 1AWD, its salt bridges are more of the long-ranged and stabilizing type of which two are placed in the core. The latter type is known to play a crucial role in the stability of proteins in general [40]. Although unique, such a lower level of salt-bridge in 1DOI may not be enough to replenish the loss of hydrophobic force under saturated salt solution.

### Extra SWs in 1DOI is related to the recognition rather than stability

Frolow et al. (1996) demonstrated excessive hydration in the surface of 1DOI that are making 40% extra hydrogen bonds with the acidic components of the N-terminal insertion domain, which they claimed as the prime reason of halo adaptation of 1DOI in supersaturated salt [39]. Our analysis also evidenced such an extra zone of SW-ATP interactions at the R1-region of 1DOI than that for 1AWD (Figure 1a). It could be possible that this peak in R1, which is in the strong hydrogen-bonding zone that is contributed by PATPs only, is related to the halo adaptation of 1DOI. However, the fact that SWs interacting at the surface is more important for the specificity and recognition than for the structural stability [6,8], we separate the buried and exposed fractions in the SW-ATP interactions. We sectioned the tertiary structure into different segments of secondary structure (i.e. H/S/C) and locations (b/e). We found, in 1DOI, the R1-region, in fact, has a lower fraction of SW-ATP interactions under buried condition. In 1DOI, in R1, the sum of normalized interactions under buried conditions is computed as: total=(26+8+11)*100/128=35.2% and that in 1AWD, as: total=(20+9+10)*100/94=41.5% (Table 4; R1). Again, for R2 and R3 regions, such types of interactions in the buried state are far less in 1DOI than that of 1AWD. This would mean that with respect to SW-PAT interactions in the interior of the protein, the halophilic signature in 1DOI is insignificant. Instead of structural stability of extra peak, we propose that the observed additional interactions may have a role in the recognition of oxido-reductase in a supersaturated salt solution.

### Core and cavity bound waters are less in 1DOI

It is known that SWs mediated bridge interactions, especially in the protein's core, play a crucial role in stabilizing the tertiary structure. It is due to the fact that in the interior, there may exist unfavorable situations related to the presence of i] isolated charge groups, ii] proximity of donor-donor or acceptor-acceptor and iii] flexibility/local-disorderness in a segment of secondary structures [6,8]. Such destabilizing situations are largely circumvented by SW mediated bridge interactions [6,8]. How much fractions of 320 moles of water per mole of 1DOI participate in such bridge interactions? Does it exceed than its mesophilic homologue (1AWD), where 126 moles of SWs are detected? These results are presented in Table 5. In this analysis, it is observed that 1DOI possesses a much lower level of P:W and W:P type bridge interactions than 1AWD. Similar is the case for a core component of these bridge interactions (Table 5; right-side column). We, therefore, inferred that the halophilic signature for core-type bridge interactions in 1DOI is insignificant with respect to its mesophilic homologue.

As far as the origin of the order waters is concerned, intuitively it can be assumed that during folding (via hydrophobic collapse), low entropic waters get trapped inside cavities. The frequency of trapped waters may depend on the inner volume of the cavities [9,35]. Because the cavities and cavity bound SWs are directly related to the stability of proteins [6,8] and because their number increases with the size of proteins, we expected these structural features in 1DOI in a much prominent manner than 1AWD. Notably, the sequence of 1DOI is longer by some 34 residues than 1AWD. Although both in 1AWD and 1DOI, the numbers of cavities are 3, the water-filled cavity in the former is more promising with respect to its contribution to the overall stability of the protein. There are two and one moles of water in an inaccessible cavity in 1AWD and 1DOI respectively with a much higher level of surrounding SWs. Further, each of these two SWs in 1AWD that are internally bound in the cavity makes a much higher level of interactions with polar and non-polar constituents (ATPs) of the cavity. In this respect, 1DOI may gain much lower stability as only one mole of water is present and that too makes the lower level of interactions. Overall, here also no special feature is seen in 1DOI that could have a direct relationship with the adaptation of protein in a hypersaline environment.

## Conclusion

We analyzed the water-ferredoxin interaction in mesophilic (moderate temperature PDB ID: 1AWD) algae and halophilic (salttolerant PDB ID: 1DOI) archaea using POWAIND version 2.0 (a protein-water interactions calculation program). It is found that the shell water (SW) is 2.5 fold greater in halophilic ferredoxin [2Fe-2S] than mesophilic ferredoxin. Water-ferredoxin interactions in the core and cavity are the signature of stability. The normalized frequency of such interactions is less in halophilic relative to mesophilic ferredoxin and the halophilic signature for stability by such interactions is negligible. However, the surface dominated with such interactions is important for ferredoxin and oxidoreductase recognition in high salt.

## Conflict of Interest

none

## Figures and Tables

**Table 1:** Database details of mesophilic and halophilic ferredoxins

Items	Mesophilic	Halophilic
Organism	Scenedesmus fuscus	Haloarcula marismortui
Protein	Plant-type ferredoxin	Plant-type ferredoxin
UniProt ID	P56408	P00217
Seq. length	94	128
RCSB ID	1AWD	1DOI
Resolution	1.4 Å	1.9 Å
Chains in str.	One (monomer)	One (monomer)
Shell-water in str.	126	320
Chromophore in str.	[2Fe-2S]	[2Fe-2S]
HELIX (DSSP)	20% helical (4H; 19R)	29% helical (7H; 38R)
SHEEL (DSSP)	30% β-sheet (7S; 29R)	25% β-sheet (7S; 32R)
Str. Structure; seq. sequence; H helices; S strands; R amino acid residues; DSSP Dictionary of Secondary Structure of Proteins [17]		

**Table 2:** Comparative analyses on sequence and structural properties of 1AWD and 1DOI

Items	1AWD	1DOI
hydrophobic	48.90%	46.90%
hydrophilic	51.10%	53.10%
Acidic and Basic	18.1% and 5.4%	26.4% and 4.6%
Aliphatic Index	74.79	81.63
pI	3.91	3.61
GRAVY	-0.19	-0.42
Sequence difference (%)	-	61.7% with 1AWD
Core composition	Core:HB 29.9%; HL 12.9%	Core:HB 29.7%; HL 15.8%
Surface composition	Surface: HB 15.1%; HL 42.7%	Surface: HB 14.2%; HL 40.5%
NCS:CS substitutions	0.66	0.42
Salt-bridges (SB)	Q=3 and types: SSs,nL, CHs,nL, CHs,nL	Q=4 and types: HHc,nL, SSc,nL, SCs,nL, HCs,nL
Net-SB stability (ΔΔG_net_)	-12.2 kcal/mol	-14.7 kcal/mol
HB hydrophobic; HL hydrophilic; NCS non-conservative; CS conservative; s surface; c core; L local; nL non-local; SS inter-strand; Q frequency; CH coil-helix; hh intra-helix; HH inter-helix; SC strand-coil		

**Table 3:** Details on normalized core and surface composition of 1AWD and 1DOI. Amino acids (single letters) are used to denote classes

	1AWD		1DOI	
Class	Co (%)	Su (%)	Co (%)	Su (%)
Hydrophobic (VILMCFAG)	29.9	15.1	29.7	14.2
Acidic (DE)	0	18.1	2.4	24.2
Basic (HRK)	0	6.5	1.6	3.9
Polar (NQSTPWY)	12.9	18.1	11.8	12.4
Total	42.8	57.8	45.5	54.7
Co core; Su surface				

**Table 4:** POWAINDv2.0 extracted range-specific frequency of ATP (Q), average temperature factor of SW (TF_av_) and average accessible surface area of ATP (ASA_av_) for SW-ATP interactions. For secondary structures, coil (C), helix (H) and strand (S) are considered. For locations, buried (b) and exposed (e) conditions are taken into account. Joint items for each ATP of Cb/Ce/Hb/He/Sb/Se-type, interacting with SW, are assessed along with TFav and ASA_av_.

	Regions	2.4-3.2Å (R1)			3.2-3.9 Å(R2)			3.9-4.2 Å (R3)		
	ATP-Type	Q	TF_av_Å^2^	ASA_av_Å^2^	Q	TF_av_Å^2^	ASA_av_Å^2^	Q	TF_av_Å^2^	ASA_av_Å^2^
1AWD	Cb	20	23	10.8	134	22	4.8	66	21.2	5.9
	Ce	52	30.2	84.1	124	27.1	120.3	70	29.2	89.2
	Hb	9	22.5	10.3	32	23.2	5.4	16	20.8	1.9
	He	22	37.7	76.1	50	28.8	85.2	25	31.1	71.7
	Sb	10	21.8	2.3	53	22.1	5.3	23	23.4	3.6
	Se	26	30.6	79.1	48	27.3	102.5	25	30.5	98.2
1DOI	Cb	26	3.1	8.2	108	4	4.7	70	4.1	5
	Ce	69	7.2	112	151	6.8	145.4	68	5.9	76.5
	Hb	8	3.5	9.7	37	3.3	6.7	23	4.6	4.7
	He	24	8.9	86.5	52	7.2	94.5	39	7.9	112.3
	Sb	11	3.6	11.6	36	3.2	4.3	25	2.8	1.8
	Se	22	6.2	67.4	51	6.3	88.5	24	5	73.2

**Table 5:** POWAINDv2.0 extracted P:W and W:P types of bridge interactions for 1AWD and 1DOI for three distance ranges (R1, R2, and R3). Each of these range-specific bridge interactions is divided into three categories such as fully exposed (e), exposed and buried (e and b) and fully buried (b) ATPs. The normalized frequencies are computed manually for each range and category. Pink shade on interacting ATPs indicates the mixed type with respect to secondary structure segments (H/S/C) along with or without mixed type location (buried/exposed i.e. b/e).

	Classes		Q (%)	Types with frequencies (%)					
e ATPs	Q %	e and b ATPs	Q %	b ATPs	Q %
1AWD (94 residues)	P:W=1:n	R1	12.8	CeCe, SeSe, HeHeε	9.6	CeCb, SeSbεε	3.2	-	0
		R2	79.8	CeCe, SeSe, HeHe, HeHeHe, SeSeSb	23.4	CeCb, SeSb, HeHb, CbCeCe, CbCbCe, SbSbSe,CbCeCbCe, HbHeHeεε	34	CbCb, SbSb,HbHb,	22.3
								CbCbCb	
									
		R3	42.6	CeCe, SeSe, HeHe, CeCeCe, SeSeSe, HeHeHe	20.2	CeCb, SeSb, HeHb, CbCeCe	9.6	CbCb,SbSb, HbHb, CbCbCb	12.8
									
	W:P=1:n	R1	8.5	CeCe, SeSe, CeHe	5.3	CeCbHbεε	1.1	HbCb,CeCbε	2.1
									
		R2	56.4	CeCe,SeSe, HeHe,CeHeHe CeCeCeε	12.8	CeCb, SbSe, HbCe, SeCb, HbHe SbCe, CbHe,HeCb, CbCbHe CbCeHeHe, CbCbCbCe	22.3	CbSb, HbSb, CbHb, SbSb, HbHbε	21.3
									
		R3	25.5	CeCe,SeCe, HeCe,CeCeCe	6.3	CeCb,SbSe,HbHe,HeCb CbHe CeHb,CbHbHe CbCbCe,SeSeSbSbCeε	14.9	SbCb, CbCb	4.2
									
1DOI (128 residues)	P:W=1:n	R1	7.8	CeCe, SeSe,HeHe	5.5	SbSe, CeCbεε	2.3	-	0
		R2	53.1	CeCe,SeSe,HeHe, CeCeCe HeHeHeHe,CeCeCeCeCeCe	21.9	CeCb, SeSb, HeHb, SbSeSe, CeCeCb, HbHbHe	22.7	CbCb, SbSb, HbHb	9.3
						CbCbCe,CbCeCbCbε			
		R3	34.4	CeCe, SeSe, HeHe,	13.3	CeCb,SeSb,HeHb, CbCbCe	14.8	CbCb, SbSb,HbHb	6.3
				SeSeSe, HeHeHe, HeHbHe					
	W:P=1:n	R1	4.7	CeCe,SeSe, CeSeε	2.3	CbCe	0.8	CbCb,HbCbε	1.6
		R2	47.7	CeCe,HeHe,SeSe,CeSe CeCeCe, SeCeCe	14.8	SeSb, HeHb, CeCb, CeHe,SbCe HeCb, CeCbCe,CbCbCe CbCeCeεε	20.3	CbCb, HbHb, SbSb, CbHb CbCbCbε	12.5
		R3	22.7	CeCe,HeHe,SeSeε	3.1	SeSb,CeCb,HeHb, CeSe, CbHe, SbHe,HeSe, HeHbCb, HbCeCe	10.9	CbCb, SbSb, CbSb	8.6
									

**Table 6:** Details of the frequency of cavity, SWs and interactions of SWs with ATPs of the cavity (Cv). Pink shade on water (W) indicates that these are present inside the cavity. Grey shade indicates TF of SWs and blue shade indicates the accessibility (Acav) of ATPs. The n is an integer. Sec_str secondary structure type; int. interactions.

	No. of protein atoms (ATPs)	No of interacting water (SWs) (≤5.0Å)		
	Cv-n (no of cavity ATPs)	Cv-1	Cv-2	Cv-3
	Cv-n→(TFav,Acav,Sec_str)	Water (no. of int.)	Water (no of int.)	Water (no of int.)
	Cv-1(16), Cv-2(9), Cv-3(8)	8 (2 inside)	3 (none inside)	4 (none inside)
1AWD	Cv-1→(18.7, 1.1, C/S/H)	W113(14), 110(10),	W107(1),W110(1), W199(1)	W107(1),W108(1)
	Cv-2→(19.0, 0.0, C)	W111(6),W114(3), W117(2),W148(2), W112(1),W121(1)		W129(1),W201(1)
	Cv-3→(20.4, 0.07, C/H)			
	Cv-1 (11), Cv-1(9), Cv-1(6)	6 (1 inside)	5 (none inside)	2 (none inside)
1DOI	Cv-1→(2.1, 0.0, C/S)	W211(8), W433(1),W304(1), W393(1),W208(3), W369(2)	W227(3),W304(2)	W310(1),W219(1)
	Cv-2→(2.9, 0.0, C)		W393(2),W424(1)	
	Cv-3→(3.3, 0.0, C)		W211(1)	

## References

[1] Anfinsen CB (1973). Science.

[2] Bandyopadhyay AK, Sonawat HM (2000). Biophys J..

[3] Bandyopadhyay AK (2001). Biochemistry.

[4] Kuntz Jr ID, Kauzmann W (1974). Adv Protein Chem..

[5] Finney JL (1977). Philos Trans R Soc Lond B Biol Sci..

[6] Ernst JA (1995). Science..

[7] Denisov VP (1997). J Phys Chem B..

[8] Williams MA (1994). Protein Science.

[9] Sterpone F (2010). J Phys Chem B..

[10] Tsodikov OV (2002). J Comput Chem..

[11] Yu B (1999). Proc Natl Acad Sci U. S. A..

[12] Royer WE (1996). Proc Natl Acad Sci U. S. A..

[13] Williams RW (1987). Biochim Biophys Acta..

[14] Bandyopadhyay AK (2007). 2007 Extremophiles.

[15] Cowan DA (1997). Comp Biochem Physiol A Physiol..

[16] Berman H (2006). Nucleic Acids Res..

[17] Rost B, Sander C (1993). J Mol Biol..

[18] Zielenkiewicz P, Saenger W (1992). Biophys J..

[19] Banerjee Bioinformation.

[20] Hall DO (1971). Nature.

[21] Tagawa K, Arnon DI (1962). Nature.

[22] Gupta PS (2014). Bioinformation.

[23] Banerjee S (2015). Bioinformation.

[24] Gupta PS (2017). Bioinformation.

[25] Gupta PS (2014). Bioinformation.

[26] Gupta PS (2015). Bioinformation.

[27] Nayek A (2015). Bioinformation.

[28] Nayek A (2015). International Journal of Institutional Pharmacy and Life Sciences.

[29] Sen Gupta PS (2017). International Journal of Engineering Science and Technology.

[30] Ikai A (1980). J Biochem..

[31] Levy Y, Onuchic JN (2006). Annu. Rev. Biophys. Biomol. Struct..

[32] Garnier J (1978). J Mol Biol..

[33] Resnik SL (1984). J Food Sci..

[34] Liang J (1998). Proteins.

[35] Rashin AA (1986). Biochemistry.

[36] Zhang L, Hermans J (1996). Proteins.

[37] Kleywegt GJ, Jones TA (1994). Acta Crystallogr D Biol Crystallogr..

[38] Lindner K, Saenger W (1978). Angew Chem Int Ed Engl..

[39] Frolow F (1996). Nat Struct Mol Biol.

[40] Nayek A (2014). Plos One.

